# Editorial: The growing problem of free-roaming dogs: a one health perspective on public and animal health

**DOI:** 10.3389/fvets.2025.1743386

**Published:** 2026-01-28

**Authors:** Gustavo Viozzi, Verónica Flores, Paula Sánchez Thevenet

**Affiliations:** 1 Laboratorio de Parasitología, Instituto de Investigaciones en Biodiversidad y Medioambiente (CONICET-Universidad Nacional del Comahue), Bariloche, Río Negro, Argentina; 2 Health Outcomes Research Group/CEU (HORG/CEU), Universidad CEU Cardenal Herrera, Castellón, Spain

**Keywords:** zoonotic diseases, human–animal interactions, dog population management, cultural perceptions, spatial epidemiology, risk perception, community engagement, zoonotic disease control

Free-roaming dogs (FRDs) represent one of the most persistent and complex global challenges at the intersection of animal welfare, public health, and environmental sustainability. Their presence in urban and rural environments affects not only biological, ecological, and epidemiological processes but also involves cultural, social, and economic issues. FRDs can act as reservoirs and transmitters of zoonotic pathogens, threaten wildlife and livestock, and pose risks to human safety and welfare; however, perceptions of dogs vary widely across societies, and influence management strategies. From a One Health perspective, addressing the problem of the growing population of free-roaming dogs is not solely a veterinary issue, but also a societal one, requiring cooperation across health, education, environmental, and policy sectors.

This Research Topic brings together contributions from different regions of the world to highlight the diversity of contexts, challenges, and solutions associated with free-roaming dogs.

In Türkiye, Pervane et al. explored the relationship between dog phobia and attitudes toward stray dog management, and they found that fear of dogs and concern about rabies significantly influenced public opinion. Their results emphasize the need for effective strategies for risk communication; emotional and psychological factors should be considered when designing public policies on dog population control. By addressing a critical gap in our understanding of how acceptance of management measures is shaped by fear and the perception of risk, the authors demonstrate that effective population control must integrate behavioral and emotional dimensions alongside epidemiological evidence.

Sarenbo and Doane focus on dog attacks on livestock in Sweden, a frequently overlooked issue compared to predation by wild carnivores. Combining data from news media, official records, and stakeholder surveys, the study shows that unsupervised dogs can cause significant economic and emotional losses to farmers; it also found that preventive measures such as mandatory leashing and enhanced monitoring could reduce conflicts and animal suffering. By documenting this neglected source of human–animal conflict, the study contributes to ongoing debates about owner responsibility, animal control legislation, and the need for evidence-based regulation within agricultural systems.

Killada et al. provide the first integrated epidemiological and diagnostic report on rabies in stray dogs in Puducherry, India, using multiple diagnostic methods and combining the monitoring of dog-bite cases with laboratory confirmation of rabies virus infection. Their findings underscore the increasing incidence of dog bites and the usefulness of alternative diagnostic tests such as LFA, dRIT, and RT-PCR, reinforcing the urgent need for sustained vaccination and public awareness programs, in line with global efforts toward the elimination of dog-mediated human rabies.

Schulte et al. examine the attitudes of pet owners in coastal Oaxaca, Mexico toward animal care and access to veterinary services. The study identifies economic and logistic impediments to responsible pet ownership and points to veterinarians as trusted community actors who could play a pivotal role in the work toward education and population control. It also emphasizes the importance of training local veterinary surgeons so as to sustain impacts that go beyond short-term sterilization campaigns. This work exposes structural inequities that limit sustainable pet care in rural Latin America, and reinforces the need for investment in veterinary training, as temporary interventions alone cannot generate lasting changes in public health or animal welfare. Particularly emphasized is the need to protect vulnerable wildlife species, like sea turtles.

The ecological dimension of FRDs is exemplified by Morucci et al., who tracked the movements of *Echinococcus granulosus*-infected dogs in rural Peru. Their results reveal alarmingly high infection rates and overlapping home ranges of infected and non-infected dogs, suggesting widespread environmental contamination between households with and without infected dogs, and a high potential for human spillover. This spatially explicit One Health approach offers valuable guidance for targeted interventions to reduce the risk of cystic echinococcosis. By integrating spatial ecology with parasitological data, this study pioneers the use of GPS tracking to aid our understanding of transmission dynamics and provide a model for geographically targeted disease-control strategies.

In another Peruvian study, Castillo-Neyra et al. document feral cave-dwelling dogs around the urban perimeter of Arequipa. These dogs have no owners and are completely unsupervised, living independently of humans. Because they are not reached by vaccination or sterilization programs, these dogs constitute a hidden reservoir that undermines any efforts to eliminate rabies. These findings call for integrated surveillance systems capable of taking these elusive populations into account when planning control strategies. This research reveals an unrecognized ecological niche that challenges conventional definitions of “free-roaming” vs. “feral” dogs, reigniting the debate about how to effectively monitor and manage these populations within One Health frameworks.

Fleming et al. provide a comprehensive review of the human dimensions and socio-ecological complexities surrounding free-roaming dogs in Australia. The authors propose a One Health-based adaptive management framework grounded in environmental psychology, incorporating typologies, audience segmentation, and interdisciplinary collaboration as tools for better governance of dog-related public and animal health issues. Their contribution advances both theoretical and applied understanding by integrating psychological and ecological perspectives, inviting renewed discussion on the balance between public safety, animal welfare, and wildlife conservation through adaptive, participatory governance.

Finally, Paredes Núñez et al. present the first large-scale serological survey of *Toxoplasma gondii* in stray dogs in Ecuador, reporting a high prevalence (39.7%) across diverse ecological regions. This study positions dogs as sentinel species for environmental contamination and advocates for integrated management action and community education to mitigate zoonotic risks. By providing novel baseline data, the research reframes dogs as bioindicators of environmental health and underscores how socio-environmental conditions influence zoonotic disease exposure in urban and rural settings.

Collectively, these studies give insight into the global scale of the FRD problem and its variability across ecological and cultural contexts, highlighting why its management remains controversial. They reveal that solutions must extend beyond veterinary interventions; education, social engagement, and culturally sensitive management strategies are all necessary. One Health approaches provide an essential framework for understanding and managing the intricate relationships between dogs, humans, and the environment. Strategies range from mass culling and confinement to sterilization and community-based management, often reflecting the tension between public safety and animal rights. Evidence from this Research Topic reinforces that isolated interventions have limited success and that integrated, One Health-driven strategies are essential. Effective long-term solutions must combine public education, vaccination, sterilization, and legislative enforcement, while respecting cultural diversity and ethical considerations. Furthermore, research must continue on how social perception, empathy, and coexistence can be leveraged to build sustainable human–animal relationships.

